# Accuracy of Real-Time Polymerase Chain Reaction in COVID-19 Patients

**DOI:** 10.1128/spectrum.00591-21

**Published:** 2022-02-16

**Authors:** Merlin Jayalal Lawrence Panchali, Hyeon Jeong Oh, You Mi Lee, Choon-Mee Kim, Misbah Tariq, Jun-Won Seo, Da Young Kim, Na Ra Yun, Dong-Min Kim

**Affiliations:** a Department of Internal Medicine, College of Medicine, Chosun University grid.254187.dgrid.464555.3 , Gwangju, Republic of Korea; b Department of Premedical Science, College of Medicine, Chosun University grid.254187.dgrid.464555.3 , Gwangju, Republic of Korea; Keck School of Medicine of the University of Southern California

**Keywords:** COVID-19, SARS-CoV-2, specificity, sensitivity, real-time polymerase chain reaction, *NP* gene, *E* gene, *RdRp*-gene

## Abstract

Coronavirus disease 2019 (COVID-19) is a mild to severe respiratory illness caused by severe acute respiratory syndrome coronavirus 2 (SARS-CoV-2). The diagnostic accuracy of the Centers for Disease Control and Prevention (CDC)- or World Health Organization (WHO)-recommended real-time PCR (RT-qPCR) primers in clinical practice remains unproven. We conducted a prospective study on the accuracy of RT-qPCR using an in-house–designed primer set (iNP) targeting the nucleocapsid protein as well as various recommended and commercial primers. The accuracy was assessed by culturing or seroconversion. We enrolled 12 confirmed COVID-19 patients with a total of 590 clinical samples. When a cutoff value of the cycle threshold (C_t_) was set to 35, RT-qPCRs with WHO RdRp primers and CDC N1, N2, and N3 primers showed sensitivity of 42.1% to 63.2% and specificity of 90.5% to 100% in sputum, and sensitivity of 65.2% to 69.6% and specificity of 65.2% to 69.6% in nasopharyngeal samples. The sensitivity and specificity of iNP RT-qPCR in sputum and nasopharyngeal samples were 94.8%/100% and 69.6%/100%, respectively. Sputum testing had the highest sensitivity, followed by nasopharyngeal testing (*P = *0.0193); self-collected saliva samples yielded better characteristics than oropharyngeal samples (*P = *0.0032). Our results suggest that iNP RT-qPCR has better sensitivity and specificity than RT-PCR with WHO (*P < *0.0001) or CDC (N1: *P = *0.0012, N2: *P = *0.0013, N3: *P = *0.0012) primers. Sputum RT-qPCR analysis has the highest sensitivity, followed by nasopharyngeal, saliva, and oropharyngeal assays. Our study suggests that considerable improvement is needed for the RT-qPCR WHO and CDC primer sets for detecting SARS-CoV-2.

**IMPORTANCE** Numerous research campaigns have addressed the vast majority of clinical and diagnostic specificity and sensitivity of various primer sets of SARS-CoV2 viral detection. Despite the impressive progress made to resolve the pandemic, there is still a need for continuous and active improvement of primers used for diagnosis in clinical practice. Our study significantly exceeds the scale of previously published research on the specificity and sensitivity of different primers comparing with different specimens and is the most comprehensive to date in terms of constant monitoring of primer sets of current usage. Henceforth, our results suggest that sputum samples sensitivity is the highest, followed by nasopharyngeal, saliva, and oropharyngeal samples. The CDC recommends the use of oropharyngeal specimens, leading to certain discrepancy between the guidelines set forth by the CDC and IDSA. We proved that the oropharyngeal samples demonstrated the lowest sensitivity for the detection of SARS-CoV-2.

## INTRODUCTION

Coronavirus disease 2019 (COVID-19), caused by severe acute respiratory syndrome coronavirus 2 (SARS-CoV-2), has a clear potential for a long-lasting global pandemic and incapacitation of health systems (1). The most common symptoms include fever, cough, and fatigue, where a few patients experience nasal congestion and rhinorrhea (2, 3). Aside from SARS-CoV-2, six major virus species that are known to cause relevant human infections includes highly pathogenic SARS-CoV and Middle East respiratory syndrome coronavirus (MERS-CoV), along with less virulent species such as NL63, 229E, OC43, and HKU1 (4). Moreover, presymptomatic and asymptomatic patients can transmit the virus and thwart the control measures, which include contact tracing and hygiene enhancement (5).

Several PCR-based techniques for detecting viral RNA have been approved as early diagnostic tests for SARS-CoV-2 infection; however, swab samples have shown varied positivity rates in PCR tests of COVID-19 patients (6, 7). A false negative diagnosis can have grave consequences, by hampering the efforts to prevent virus transmission (8). The efficiency of detection by an IgM enzyme-linked immunosorbent assay (ELISA) is higher than that of real-time PCR (RT-qPCR) at 5.5 days after symptom onset, as reported in one study (9). The gold standard diagnosis is believed to require a positive culture and has over a 4-fold increase in a SARS-CoV-2 antibody titer (10, 11). Meanwhile, few studies have indicated that self-collected saliva samples can be used for diagnosing COVID-19 (12, 13). To date, only a few studies have shown that which samples may have the highest potential for the accurate diagnosis of COVID-19.

In this study, we validated the diagnostic sensitivity and specificity of RT-qPCR–based assays that identify the three major genes of SARS-CoV-2 in patients with clinical and subclinical COVID-19. Furthermore, we explored the diagnostic specificity and sensitivity of RT-qPCR in the upper- and lower-respiratory-tract samples, self-collected saliva samples, and other samples collected from the patients. So far, no prospective studies have proven the specificity and sensitivity of the Centers for Disease Control and Prevention (CDC) or World Health Organization (WHO) recommended RT-qPCR primers and evaluated their accuracy in clinical practice. In this study, we determined the specificity and sensitivity of different RT-qPCR primer sets, including the CDC and WHO primers, to assess and compare their accuracy.

## RESULTS

### Clinical characterization of patients.

All patients were admitted and treated at a single tertiary hospital (Chosun University Hospital, Gwangju, South Korea). Between February 21, 2020 and May 11, 2020, we enrolled 12 confirmed COVID-19 patients. The median age of all the patients was 49.16 years (range, 22 to 79 years), including seven men and five women. Two of these patients were asymptomatic and did not have any clinical signs or symptoms of COVID-19 throughout the study period. In addition to the confirmed patients, 107 healthy subjects were enrolled, and 128 SARS-CoV-2-negative samples were collected in this study (Table S2). The clinical data for the patients with COVID-19 are listed in Table S3.

### *In vitro* RT-PCR analysis of other respiratory viruses and bacteria for cross-reactivity assessment.

Numerous respiratory-disease viruses and bacterial strains were assayed by RT-qPCRs with all the primers evaluated in this study. The *E* gene primer set of the Kogene Kit, the WHO *RdRp* primer set, and the CDC *N2* and *N3* primer set showed cross-reactivity with the purified SARS-CoV Urbani strain. Whereas WHO *RdRp* primer showed a cross-reactivity with influenza A virus with higher C_t_ value. However, the CDC N1 and N2 primer sets had cross-reactivity with influenza A, influenza B, and influenza C virus with C_t_ values above 35. Moreover, the reliability of the CDC *N1* primer set differed from that of the other primer sets when the C_t_ value was set above >35. These data suggested that RT-PCR with either the iNP primer set or the *RdRp* primer set (Kogene Kit) has better specificity for the SARS-CoV-2 virus. The results are summarized in Table 1.

**TABLE 1:** RT-qPCR results on other respiratory viruses and bacteria^*a*^

Type	Virus/bacteria strain name	RT-qPCR results of different target genes and primer sets (cycle threshold)						
		NP-gene (iNP)	E-gene (Kogene kit)	RdRp-gene (Kogene kit)	WHO RdRp primers	CDC N1 primers	CDC N2 primers	CDC N3 primers
Virus	Avian infectious bronchitis virus, strain *Massachusetts*	ND	ND	ND	ND	35.84	ND	ND
Virus	Human *Coronavirus* NL63	ND	ND	ND	ND	38.47	ND	ND
Virus	Canine *coronavirus* Strain UCD1	ND	ND	ND	ND	37.04	ND	ND
Virus	*MERS-CoV*	ND	ND	ND	UD	ND	ND	ND
Virus	*SARS-CoV* Purified, in PBS 1 × 10^8 pfu/mL (eq), Urbani strain	ND	24.23	ND	31.76	38.08	30.10	25.36
Virus	*Human respiratory syncytial virus*, Strain A2000/3-4	ND	ND	ND	ND	36.47	38.95	ND
Virus	*Influenza A/Texas/36/91, H1N1*	ND	ND	ND	35.51	37.55	38.57	ND
Virus	*Influenza B/Florida/4/2006*	ND	ND	ND	ND	ND	37.81	ND
Virus	*Influenza C virus C/Taylor/1233/1947*	ND	ND	ND	ND	39.17	39.03	ND
Virus	*Measles virus* Edmonston	ND	ND	ND	ND	ND	39.02	ND
Virus	*Rhinovirus*	ND	ND	ND	ND	ND	ND	ND
Virus	Human astrovirus (HAstV) type 1	ND	ND	ND	ND	ND	ND	ND
Virus	Human astrovirus (HAstV) type 2	ND	ND	ND	ND	36.34	ND	ND
Bacteria	Klebsiella pneumoniae Isolate 1	ND	ND	ND	ND	38.70	ND	ND
Bacteria	Klebsiella oxytoca MIT 10-5244	ND	ND	ND	ND	37.16	ND	ND
Bacteria	Leptospira interrogans HAI0156 (Serovar Copenhageni)	ND	ND	ND	ND	37.13	ND	ND
Bacteria	Mycobacterium abscessus #103	ND	ND	ND	ND	37.37	ND	ND
Bacteria	Mycobacterium avium 2285 Smooth	ND	ND	ND	ND	37.10	ND	ND
Bacteria	Mycobacterium intracellulare 1956	ND	ND	ND	ND	37.30	ND	ND
Bacteria	Staphylococcus aureus Strain AIS 1000505 AKA VRS10	ND	ND	ND	ND	37.73	ND	ND
Bacteria	Staphylococcus*; aureus* MRSA; M0200	ND	ND	ND	ND	37.02	ND	ND
Bacteria	Streptococcus pneumoniae Strain TCH8431	ND	ND	ND	ND	ND	ND	ND
Bacteria	Pseudomonas aeruginosa ATCC 27853	ND	ND	ND	ND	ND	ND	ND

a ND, not detectable; cycle threshold values are presented as obtained here. Cycle threshold >35 is considered a negative result for the Kogene Kit; iNP, the in-house–designed primer set targeting the *NP* gene.

### Evaluation of SARS-CoV-2 via RT-PCR.

In total, 590 various clinical samples, including nasopharynx (17.14%), oropharynx (15.25%), sputum (17.96%), saliva (11.69%), urine (15.59%), stool (9.32%), serum/plasma, and whole blood (13.05%) samples were analyzed for the presence of the viral RNA of SARS-CoV-2, as summarized in Table S4. For the 12 patients, the viral load in the samples starting from the onset of symptoms to the recovery phase was analyzed by RT-qPCR. The earliest sample was collected 2 days prior to symptom onset, and the latest sample was taken on the 74th day post-recovery. Almost all patients’ swabs tested positive for up to 7 days after symptom onset. Viral shedding from nasopharyngeal samples was substantially higher than that from oropharyngeal samples during the early stage of symptoms, from day 0 to day 7 (*P = *0.006); coinciding with the detection of viral RNA up to day 14 to 15 in nasopharyngeal samples but only up to 7 to 8 days in the oropharyngeal samples at a C_t_ cutoff of 35 (C_t_-35). In sputum samples, we consistently detected viral RNA with low C_t_ values. In two patients, viral RNA was detectable on 34th and 64th day of sputum sample collection (data not shown).

Simultaneously, RT-qPCR with the iNP primer set was performed for all the samples collected in this study. In agreement with previously published data (14), we were able to detect SARS-CoV-2 RNA in only five (6.32%) samples from the 77 blood samples collected during the entire study period. Of the 101 nasopharyngeal and 90 oropharyngeal samples collected, 42 (44.68%) and 21 (23.59%) samples tested positive for SARS-CoV-2 in iNP RT-PCR, respectively. Among the 106 sputum samples, 61 (57.54%) samples tested positive for this virus. To identify the presence of viral RNA in saliva, 69 samples were collected, of which 32 (46.37%) tested positive. Only one (1.08%) urine sample tested positive out of the 92 urine samples collected, and only six (10.90%) out of 55 stool samples tested positive for SARS-CoV-2 in iNP RT-qPCR at C_t_-35. The results are summarized in Table S4.

To determine the accuracy of iNP RT-qPCR, we cross-checked all the samples with the Kogene Kit targeting the *E* and the *RdRp* gene. For rigorous quality standards as well as for cost effectiveness, we selected only nasopharyngeal and sputum samples to continue our evaluation of the specificity and sensitivity of other primer sets such as the WHO primers, CDC primers, and Kogene Kit.

### Diagnostic specificity and sensitivity in samples up to 3 days after admission and a comparison with various RT-qPCR primer and probe sets.

To scrutinize diagnostic specificity and sensitivity and to examine all the primer sets targeting SARS-CoV-2, we selected the nasopharyngeal and sputum samples collected between days 0 to 3 after hospital admission. We also attempted to determine differences in cutoffs, wherein we chose 35 and 40 as the cutoff for all RT-qPCR primers. When the cutoff C_t_-35 was used, the sensitivity/specificity of iNP RT-qPCR in sputum was 94.8%/100%, and in nasopharyngeal samples, it was 69.6%/100%, respectively. RT-qPCRs with the *E* gene and *RdRp* gene primer sets (Kogene Kit) had sensitivity and specificity of 84.2%/100% and 94.8%/100% in sputum samples and 60.9%/100% and 60.9%/100% in nasopharyngeal samples, respectively (Table 2). In contrast, RT-qPCR with the WHO RdRp primer set manifested a sensitivity/specificity of 42.1%/100% in sputum samples and 65.2%/100%, respectively, in nasopharyngeal samples. On the other hand, the RT-PCRs with CDC *N1*, *N2*, and *N3* primers had a sensitivity and specificity of 69.6%/100%, 65.2%/96.4%, and 69.6%/100% in nasopharyngeal samples and 57.9%/100%, 63.2%/90.5%, and 57.9%/100% in sputum samples, respectively, as summarized in Table 2.

**TABLE 2:** Sensitivity and specificity of RT-qPCR analysis of patients’ samples from the day of admission to the third day, for various primers and probes^*a*^

Primer sets	Sputum samples		Nasopharynx samples	
	Ct-35	Ct-40	Ct-35	Ct-40
NP-gene (iNP) Sensitivity/Specificity (AUC)	94.8%/100% (0.97)	100% /100% (1.0)	69.6% /100% (0.82)	73.9% /100% (0.87)
E-gene (Kogene kit) Sensitivity/Specificity (AUC)	84.2% /100% (0.92)	89.5% /100% (0.95)	60.9% /100% (0.80)	60.9% /100% (0.80)
RdRp-gene (Kogene kit) Sensitivity/Specificity (AUC)	94.8%/100% (0.97)	100% /100% (1.0)	60.9% /100% (0.83)	65.2% /100% (0.79)
WHO RdRp primers Sensitivity/Specificity (AUC)	42.1%/100% (0.71)	79.0%/100% (0.90)	65.2% /100% (0.82)	86.4% /96.4% (0.90)
CDC N1 primers Sensitivity/Specificity (AUC)	57.9%/100% (0.79)	89.5%/90.5% (0.86)	69.6% /100% (0.85)	82.6% /85.7% (0.84)
CDC N2 primers Sensitivity/Specificity (AUC)	63.2%/90.5% (0.73)	73.7%/100% (0.79)	65.2% /96.4% (0.79)	60.3% /96.4% (0.67)
CDC N3 primers Sensitivity/Specificity (AUC)	57.9%/100% (0.79)	89.5%/100% (0.95)	69.6% /100% (0.85)	73.9/100% (0.87)

a Number of SARS-CoV-2–positive sputum samples = 19; number of virus-negative sputum samples = 21; number of virus-positive nasopharyngeal samples = 23; number of virus-negative nasopharyngeal samples = 28; iNP, in-house–designed *NP* gene primer set; C_t_-35, cutoff cycle threshold of 35; C_t_-40, cutoff cycle threshold of 40.

**FIG 1:**
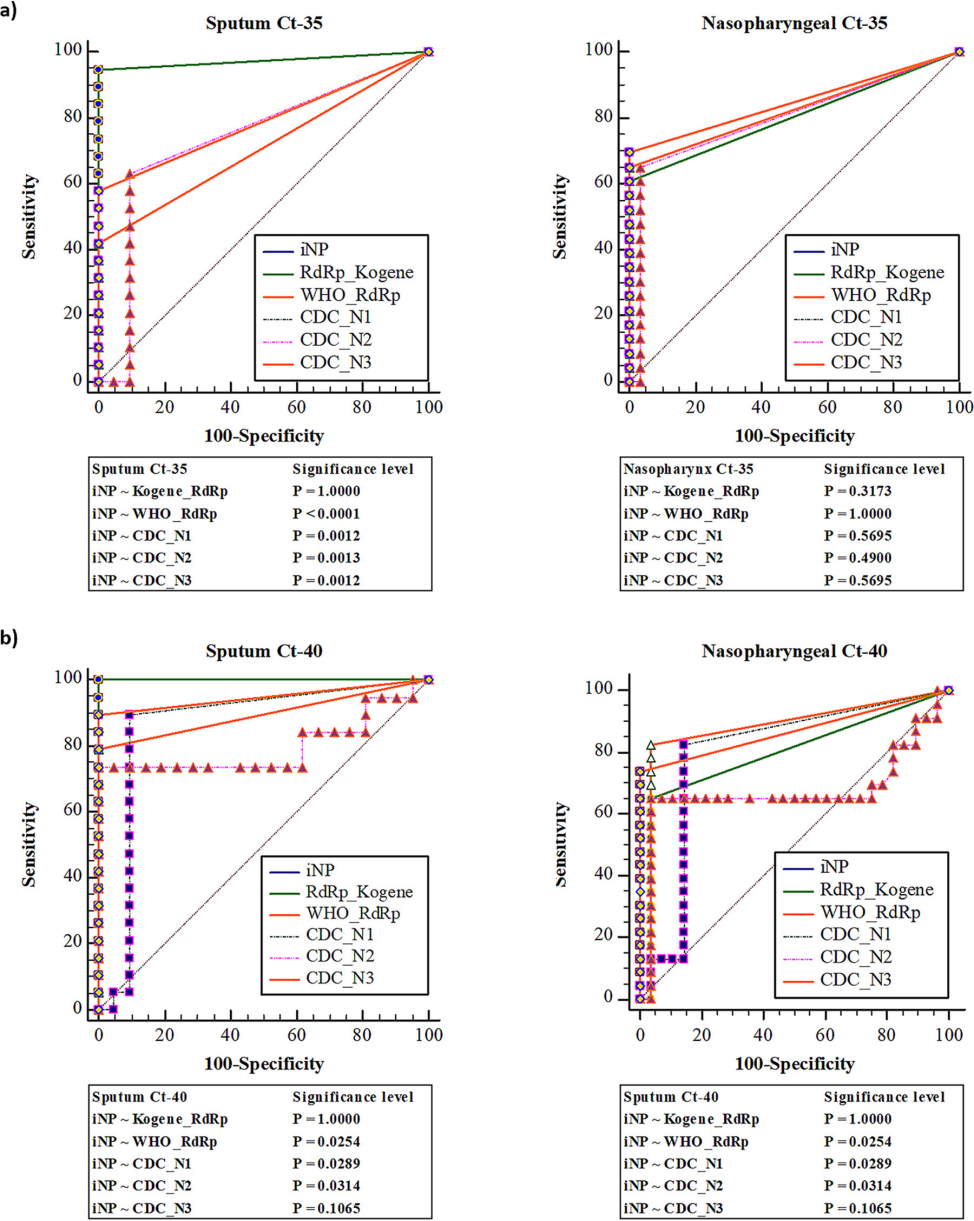
Comparison of the specificity and sensitivity of various commercial RT-qPCR primer sets among selected clinical samples. (a) Evaluation of specificity and sensitivity in sputum and nasopharyngeal samples at a C_t_ cutoff of 35 (C_t_-35). (b) Evaluation of specificity and sensitivity in sputum and nasopharyngeal samples at a C_t_ cutoff of 40 (C_t_-40).

When C_t_ was set to 40, a slight increase in sensitivity was observed in RT-qPCR involving either the iNP primers or Kogene Kit primers, whereas RT-qPCR with the primers recommended by the WHO and CDC showed significantly varied specificity and sensitivity (Fig. 1).

In brief, RT-qPCR using the primers for either the iNP assay or the *RdRp* gene (Kogene Kit) had the highest sensitivity (94.8%, C_t_-35) in the sputum samples as compared with all the other primers. Nevertheless, in the nasopharyngeal samples, the sensitivity of iNP at C_t_-35 was 69.9%, and the sensitivity of the CDC primers targeting genes *N1* (69.6%), *N2* (65.2%), and *N3* (69.6%) were comparable, as presented in Table 2. All primer sets were 100% specific in both the sputum and nasopharyngeal samples, with the exception of the *N2* gene (90.5% specificity in sputum and 96.4% in nasopharynx samples), respectively. Hence, RT-qPCR with the iNP primers was superior to RT-qPCR involving the (target gene *E*) Kogene Kit primers (iNP versus *E* gene: *P* = 0.014 in sputum, and iNP versus *E* gene: *P* = 0.056 in nasopharyngeal samples) when considering sensitivity in both sputum and nasopharyngeal samples (Table 2). Our results suggest that for the detection of SARS-CoV-2, sensitivity in sputum samples is significantly higher than that in nasopharyngeal samples and is more suitable for PCR-based diagnosis (C_t_-35, *P = *0.0193; C_t_-40, *P = *0.0012; Fig. S1a, b).

### Analysis of the specificity and sensitivity of RT-qPCR depending on the duration of COVID-19.

To determine changes with time, we further analyzed the specificity and sensitivity of the samples collected from the patients depending on the time interval from the date of admission to complete recovery. The samples at different time points from the date of admission, namely, at 0 to 3 days, 1 week, 2 weeks, 3 weeks, 4 weeks, and 5 or more weeks, were analyzed for specificity and sensitivity (Table 3).

**TABLE 3:** RT-qPCR specificity and sensitivity in sputum versus nasopharyngeal samples from symptom onset to recovery^*a*^

Days since symptoms	Sputum (*N* = 101)						Nasopharynx (*N* = 106)					
	Ct-35			Ct-40			Ct-35			Ct-40		
	Design	Kogene Kit		Design	Kogene Kit		Design	Kogene Kit		Design	Kogene Kit	
	NP gene	E gene	RdRP	NP gene	E gene	RdRP	NP gene	E gene	RdRP	NP gene	E gene	RdRP
Admission date Sensitivity (%)/Specificity (%) (AUC)	80.0/100 (0.90)	70.0/100 (0.85)	70.0/100 (0.85)	100/100 (1.0)	90.0/100 (0.95)	90.0/100 (0.95)	66.7/100 (0.83)	66.7/100 (0.83)	66.7/100 (0.83)	66.7/100 (0.83)	83.3/100 (0.92)	75.0/100 (0.87)
0 to 3 days Sensitivity (%)/Specificity (%) (AUC)	86.4/100 (0.93)	81.8/100 (0.91)	81.8/100 (0.91)	90.9/100 (0.95)	95.4/100 (0.98)	95.4/100 (0.98)	65.0/100 (0.83)	70.0/100 (0.85)	65.0/100 (0.83)	65.0/100 (0.83)	75.0/100 (0.86)	70.0/100 (0.85)
1 wk (0 to 7 days) Sensitivity (%)/Specificity (%) (AUC)	83.9/100 (0.92)	77.4/100 (0.89)	80.6/100 (0.90)	87.1/100 (0.93)	90.3/100 (0.95)	90.3/100 (0.95)	73.5/100 (0.87)	70.6/100 (0.85)	70.6/100 (0.85)	79.4/100 (0.90)	79.4/100 (0.90)	76.5/100 (0.88)
2 wks (8 to 14 days) Sensitivity (%)/Specificity (%) (AUC)	72.0/100 (0.86)	52.0/100 (0.76)	52.0/100 (0.76)	84.0/100 (0.92)	72.0/100 (0.86)	72.0/100 (0.86)	47.8/100 (0.74)	34.8/100 (0.67)	34.8/100 (0.67)	52.2/100 (0.76)	52.2/100 (0.76)	52.2/100 (0.76)
3 wks (15 to 21 days) Sensitivity (%)/Specificity (%) (AUC)	42.1/100 (0.71)	21.1/100 (0.60)	21.1/100 (0.60)	47.4/100 (0.74)	36.8/100 (0.68)	47.4/100 (0.74)	17.7/100 (0.59)	5.9/100 (0.53)	5.9/100 (0.53)	23.5/100 (0.62)	29.4/100 (0.65)	23.5/100 (0.62)
4 wks (22 to 29 days) Sensitivity (%)/Specificity (%) (AUC)	50.0/100 (0.75)	50.0/100 (0.75)	33.3/100 (0.67)	83.3/100 (0.92)	50.0/100 (0.75)	50.0/100 (0.75)	0/100 (0.50)	0/100 (0.50)	0/100 (0.50)	0/100 (0.50)	0/100 (0.50)	0/100 (0.50)
5 wks+ (30 + days) Sensitivity (%)/Specificity (%) (AUC)	15.8/100 (0.58)	5.3/100 (0.53)	5.3/100 (0.53)	42.1/100 (0.71)	26.3/100 (0.63)	15.8/100 (0.58)	7.1/100 (0.54)	0/100 (0.50)	0/100 (0.50)	21.4/100 (0.61)	7.1/100 (0.54)	14.3/100 (0.57)

a Samples were segregated as follows: admission date; 0 to 3 days after admission; 0 to 7 days after admission as 1 week; 8 to 14 days as 2 weeks; 15 to 21 days as 3 weeks; 22 to 29 days as 4 weeks; and 30+ days as 5 weeks+. *N*, number of samples; C_t_-35, cycle threshold (cutoff) of 35; C_t_-40, cycle threshold (cutoff) of 40. All samples were collected between days 0 and 3 after symptom onset.

To compare the results, we examined the specificity and sensitivity with both cutoff values, C_t_-35 and C_t_-40. As expected, the sensitivity of iNP RT-qPCR was significantly higher than that of the primers targeting genes *E* and *RdRp* in the Kogene Kit when the C_t_ cutoff was 35. Meanwhile, the positivity rate of sputum samples was much higher than that of nasopharyngeal samples, as illustrated in Table 3. In the analysis of sensitivity depending on C_t_, the sensitivity of iNP RT-qPCR at C_t_-35 was significantly higher than that of the Kogene Kit. On the other hand, when the C_t_ cutoff was 40, the sensitivity markedly increased for both target genes *E* and *RdRp* of the Kogene Kit. This finding may be due to nonspecific bands similar to those reported in another study (14), because the manufacturer (Kogene) recommends a C_t_ cutoff of 35, our hypothesis of nonspecificity may be valid (Fig. 1 and Table 3).

### Comparison of the specificity and sensitivity of iNP RT-qPCR among sputum, nasopharyngeal, saliva, and oropharyngeal samples.

**FIG 2:**
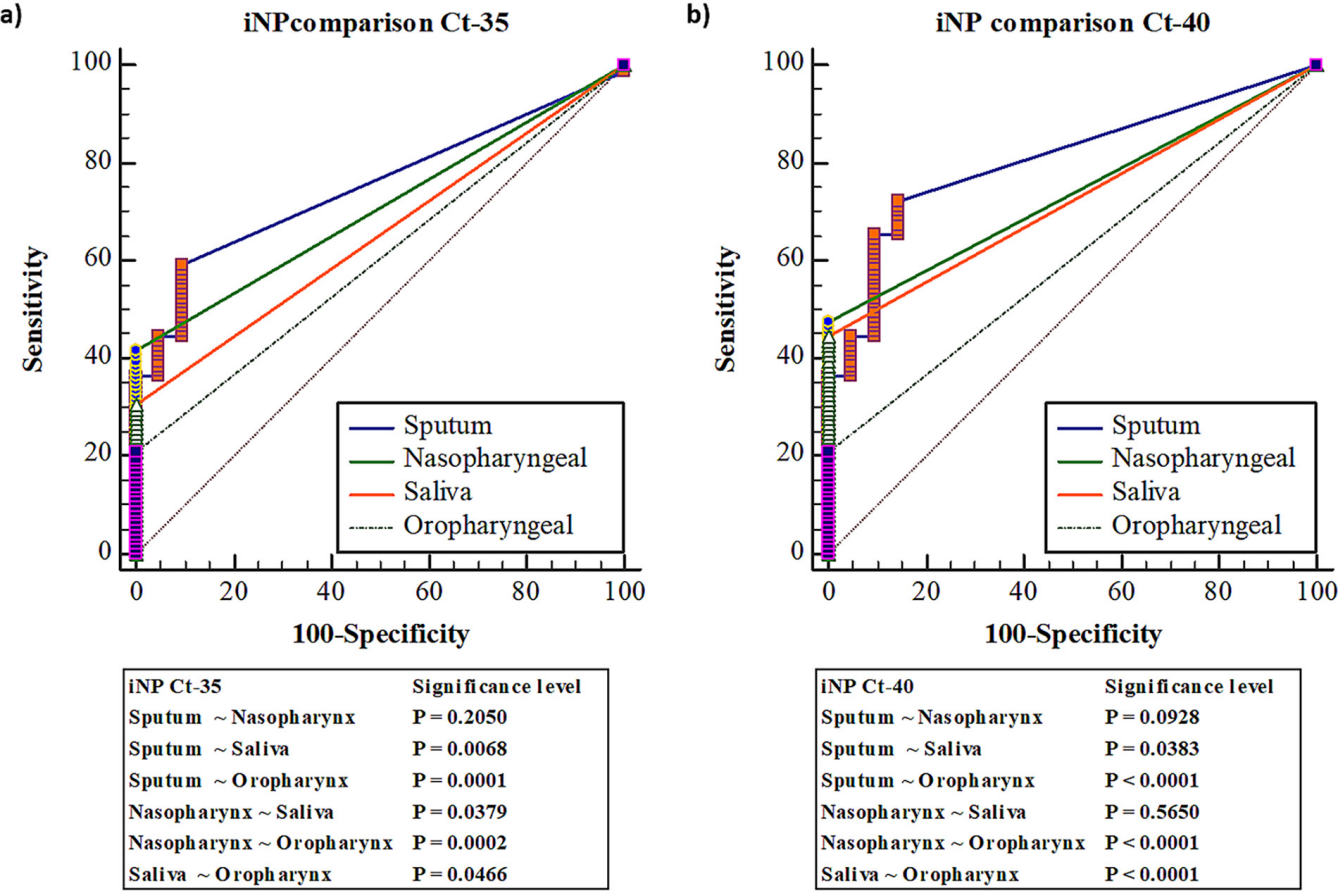
Comparison of the specificity and sensitivity of RT-qPCR involving the in-house–designed *NP* gene primer set (iNP) among sputum, nasopharyngeal, saliva, and oropharyngeal samples. (a) Determination and comparison of the specificity and sensitivity of iNP RT-qPCR among sputum, nasopharyngeal, saliva, and oropharyngeal samples at a C_t_ cutoff of 35 (C_t_-35). (b) Determination and comparison of the specificity and sensitivity of iNP RT-qPCR among sputum, nasopharyngeal, saliva, and oropharyngeal samples at a C_t_ cutoff of 40 (C_t_-40).

We analyzed and compared the specificity and sensitivity of iNP RT-qPCR among various samples (nasopharyngeal, oropharyngeal, and saliva samples) at C_t_ cutoffs of 35 or 40. A more significant difference was observed between the saliva and nasopharyngeal samples (*P = *0.0379, Fig. 2a) samples. Meanwhile, sensitivity in saliva was significantly higher than that in oropharyngeal samples at C_t_-35 (*P = *0.0032) during the first week after symptom onset (Fig. S1c, d). Our results indicated that sensitivity was highest in sputum samples, followed by nasopharyngeal, saliva, and oropharyngeal samples, as illustrated in Fig. 2a and b. Thus, sputum samples can be considered the primary clinical material for COVID-19 diagnosis. Unfortunately, we cannot eliminate the risk of aerosolization of virus particles while collecting a sputum samples.

## DISCUSSION

A few authors have reported diagnostic accuracy in confirmed COVID-19 patients along with serological and culture-based results (15–17); however, major differences in analytical specificity and sensitivity among the samples of different human tissues/biological liquids and among time points after the onset of SARS-CoV-2 have not been addressed. Our results revealed that the most available primer sets need to be reassessed regarding their specificity and sensitivity because they can yield false negative and false positive results and therefore an incorrect diagnosis. SARS-CoV-2 infection is widespread in family clusters, on food premises, at workplaces, and religious gatherings owing to the presence of high viral load in a patient at the initial stage of infection, even if there are relatively mild or no symptoms (18, 19).

Present day diagnostic methods are based on RT-qPCR or deep-sequencing technologies that require some replication of a viral RNA to ensure that a sufficient amount of the virus is collected for diagnosis (20). Under the present scenario, viral RNA detection by RT-qPCR is regarded as one of the principal diagnostic methods for COVID-19 (21). Nevertheless, the reliability of RT-qPCR has been debated due to false negative and false positive results (22). In some cases, positive results are confirmed after a full recovery or in the absence of infection; for some patients, the COVID-19 diagnosis has been falsely ruled out based on consecutive negative results of RT-qPCR analysis of respiratory-tract samples (23). In other cases, the patients were suspected to be SARS-CoV-2–positive according to clinical presentation and a history of exposure to the disease, but their oropharyngeal and nasopharyngeal swabs repeatedly tested negative in RT-qPCR. Eventually, bronchoalveolar lavage fluid was found to be SARS-CoV-2–positive in RT-qPCR on the eighth day (24).

A few studies have assessed self-collected saliva and other samples for diagnosing COVID-19; however, to date, the selection of clinical samples for accurate diagnosis has not been specified (25, 26). In the present study, 31 saliva samples tested positive, with a sensitivity of 79.1% in the first week and a drastic decline to 37.5% at 2 weeks after symptom onset. None of the saliva samples collected after 3 weeks tested positive throughout our study. Our data were consistent with previously published results, where the high viral load was reported in severe cases and persisted for a long time in clinical samples, whereas, in mild cases of COVID-19, high viral load is detectable at the initial stage and can disappear at a later stage (27).

One study suggested that the rate of SARS-CoV-2 positivity in sputum samples is significantly higher than that for throat swabs, and sputum samples may be of greater significance for diagnosing COVID-19 (28). However for patients with early-stage infection, especially for asymptomatic or mild cases, nasopharyngeal samples yield more reliable results. The nasopharyngeal samples showed a sharp decline in RT-qPCR sensitivity within 1 week, whereas sensitivity in sputum samples was consistent for ∼2 weeks and gradually declined thereafter. The Infectious Diseases Society of America (IDSA) suggests the use of nasopharyngeal, mid-turbinate, or nasal samples rather than oropharyngeal or saliva samples for COVID-19 diagnosis (29). However, the suitability of upper-respiratory-tract samples remains uncertain (29). The CDC recommends the use of oropharyngeal specimens collected by a health care professionals, leading to certain discrepancy between the guidelines set forth by the CDC and IDSA (30). In our study, we proved that the oropharyngeal samples demonstrated the lowest sensitivity for the detection of SARS-CoV-2. A recent study provided evidence that in the detection of SARS-CoV-2, saliva samples may yield higher sensitivity than nasopharyngeal samples (31). Moreover, the present study revealed that iNP RT-qPCR analysis of self-collected saliva samples has higher sensitivity than oropharyngeal samples as well as during the first week after symptom onset. Although our study evaluated the sensitivity and specificity of various clinical samples with various primer sets targeting the SARS-CoV-2 genome, the study did have limitations which included: the small sample size, and the lack of viral load and culture data on daily basis. In addition to the assay of sensitivity and specificity, we revised the PCR conditions of Roche master with a gradient PCR in order to optimize the CDC Primers assay according to our laboratory instrumentation (27).

### Conclusion.

Our study shows the analytical and diagnostic specificity and sensitivity of various RT-qPCR primers used in clinical practice. Sputum samples yielded the highest sensitivity of RT-qPCRs, followed by nasopharyngeal, saliva, and oropharyngeal samples in patient’s diagnosis with COVID-19. Moreover, we report evidence that the CDC and WHO primers require considerable improvement for more accurate detection of SARS-CoV-2.

## MATERIALS AND METHODS

### Participants.

We conducted a prospective cohort study on confirmed COVID-19 patients from February 21, 2020 to May 11, 2020 at Chosun University Hospital, South Korea. The participants were confirmed to be SARS-CoV-2 positive by diagnostic methods such as RT-qPCR, cell culture and a >4-fold increase or seroconversion in terms of the SARS-CoV-2 antibody titer. Negative controls were obtained from healthy subjects with no clinical symptoms, no history of contact with confirmed COVID-19 patients, and no history of antibody detection of a SARS-CoV-2.

### Sampling and RNA extraction.

Nasopharynx and oropharynx swabs, sputum, saliva, urine, stool, serum/plasma, and whole blood were collected, and 200 μL of each sample was used for RNA extraction. Sputum, saliva, stool, and urine samples were self-collected by the patients. Sputum, saliva, and stool samples were diluted in phosphate-buffered saline (PBS), mixed, and centrifuged, and the supernatant was subjected to RNA extraction. Nasopharynx and oropharynx swabs were collected by a physician directly to the commercial UTM kits containing 1 mL of a viral transport medium (Noble Bio, Korea) and were employed for RNA extraction. The viral RNA was extracted by a fully automated instrument (Real-Prep system, Biosewoom, South Korea) with the Real-prep Viral DNA/RNA Kit (Biosewoom, South Korea).

### Cell culture.

Vero E6 cells were used for the culturing and identification of SARS-CoV-2. All cell culture work of SARS-CoV-2 virus was performed in a laboratory with Biosafety Level 3 (BL3) facility (Health and Environment Research Institute of Gwangju City). The cells were cultured in Dulbecco’s modified Eagle’s medium (DMEM) supplemented with 10% fetal bovine serum and a 1× penicillin–streptomycin antibiotic solution (Gibco, Thermo Fisher Scientific Inc., Korea) in an atmosphere containing 5% CO_2_ at 37°C. The swab samples were obtained by means of the UTM kit with 1 mL of the viral transport medium (Noble Bio, Korea), or the samples collected into collection tubes were diluted with 1 mL of Dulbecco’s phosphate-buffered saline (Welgene, Korea) and thereafter inoculated into a monolayer of the cultured Vero cells. The inoculated culture was examined daily for cytopathic effects, similar to the procedures used for SARS-CoV and MERS-CoV in other studies (32, 33).

### The enzyme-linked immunosorbent assay.

Serological assays, including tests for IgG, IgM, and total antibody (IgG, IgM, and IgA) titers, were performed using an indirect ELISA. Briefly, each well of 96-well ELISA microplates (Thermo Fisher Scientific, Waltham, MA, USA) was coated with 100 μL of 2 μg/mL plant recombinant SARS-CoV-2 nucleocapsid protein (BIOAPP. Inc., Korea) in carbonate-bicarbonate buffer followed by overnight incubation at 4°C. The microplates were washed with PBS supplemented with 0.05% Tween 20 (PBS-T) and blocked 5% skim milk in PBS-T for 2 h at 37°C. After washing, the serum samples were diluted 100-fold with blocking buffer and incubated at 37°C for 2 h. The plates were rewashed, and a secondary antibody (HRP-conjugated goat anti-human IgG antibody [1:6,000 Invitrogen, Thermo Fisher Scientific, Cat A18805], an anti-human IgM antibody [1:3,000 Invitrogen, Thermo Fisher Scientific, Cat 31415], or an anti–human-total-antibody [1:40,000; Thermo Fisher Scientific, Cat 31418]) was added, and the plate was incubated again at 37°C for 1 h. After washing, 50 μL of the 3,3′5,5′-tetramethylbenzidine substrate (TMB, Sigma-Aldrich, St. Louis, MO, USA) was added at room temperature and incubated for 30 min in dark. The reaction was stopped with 25 μL of 1 M H_2_SO_4_, and optical density at 450 nm (OD_450_) was measured. The cutoff values were determined by calculating the mean OD_450_ plus 3-fold standard deviation of the negative serum samples. Thus, the observed cutoffs for IgG, IgM, and total antibodies were 1.1, 0.5, and 0.7, respectively. When the OD of a patient sample was greater than the calculated cutoff, it was considered positive for SARS-CoV-2.

### The indirect immunofluorescent assay.

The SARS-CoV-2 virus was obtained from the Korea Disease Control and Prevention Agency and was used to infect Vero E6 cells. To prepare a SARS-CoV-2 antigen slide, cells infected for 3 days were cultured on Teflon-coated multiwell slides overnight at 37°C and 5% CO_2_ and were fixed with 80% acetone the next day. Each patient’s serum was subjected to 2-fold serial dilutions starting from 1:16 and then reacted with SARS-CoV-2 viral antigens in a moist chamber for 30 min at 37°C. After washing, the slides were further incubated with a 1:400-diluted secondary antibody (a fluorescein isothiocyanate–conjugated anti-human IgM or IgG antibody; MP Biomedicals, OH, USA). Then, the slides were observed under a fluorescence microscope (Olympus IX73, magnification: 400×) after the addition of mounting solution (VECTOR Laboratories, Burlingame, CA 94010, USA) on the slides. An IgG antibody titer ≥1:32 was chosen as the cutoff for the indirect immunofluorescent assay (IFA) using the clinical samples from 15 healthy individuals (34).

### RT-qPCR for SARS-CoV-2 detection.

RT-qPCR was performed targeting the *NP*, *E*, and *RdRp* genes along with a reference gene as a positive control and distilled water as a negative control. For the RT-qPCR assay of the *NP* gene, primers and probes were designed in-house (iNP assay), whereas for target genes *E* and *RdRp*, the Kogene Kit (Kogene Biotech Seoul, South Korea) were utilized, and the amplification was carried out according to the manufacturer’s specifications. For iNP target, RT-qPCR was performed in Exicycler 96 Real-Time Quantitative Thermal Block (Bioneer, Daejeon, Korea), and for Kogene kits CFX96 Touch Real-Time PCR Detection System (CA, USA) was used. RT-qPCR optimization with Roche master mix (LightCycler Multiplex RNA Virus Master) was conducted with a gradient RT-qPCR and the conditions we set for optimal results. Cycle threshold (C_t_) values were set to ≤35 and ≤40 for the reference gene and were assumed to denote a positive result. C_t_ values >35 were assumed to indicate a negative result. To compare the specificity and sensitivity of these primer sets with those of other neutral primers, we chose the primers recommended by the CDC and WHO. All primer details are listed in Table S1.

### Statistical methods.

Sensitivity, specificity, and positive and negative predictive values and accuracy were expressed as percentages. Quantitative data were expressed as the mean ± standard deviation or as the median (range) and percentage (95% confidence interval). Categorical variables were compared using either the chi-square test or Fischer’s exact test, when appropriate. Continuous variables were compared using the Mann–Whitney nonparametric test, when appropriate. Statistical significance was assumed at *P* values <0.05. The mean data were used to evaluate sensitivity and specificity via the area under the receiver operating characteristic (ROC) curve. All statistical analyses were performed using the MedCalc software (Ostend, Belgium) (35).
