# Supplementary material for: Accuracy of Real-Time Polymerase Chain Reaction in COVID-19 Patients
Source: Microbiol Spectr. 2022 Feb 16;10(1):e00591-21. doi: 10.1128/spectrum.00591-21 (PMC8849071; doi:10.1128/spectrum.00591-21)
Supplement: SUPPLEMENTAL FILE 1 — Supplemental material. Download SPECTRUM00591-21_Supp_1_seq4.pdf, PDF file, 0.7 MB [file spectrum00591-21_supp_1_seq4.pdf]

Supplementary Materials

Supplementary Figures

Figure S1. The specificity and sensitivity of RT-qPCR with the in-house–designed *NP* gene primer set (iNP) in nasopharyngeal vs. sputum samples and oropharyngeal vs. saliva samples.

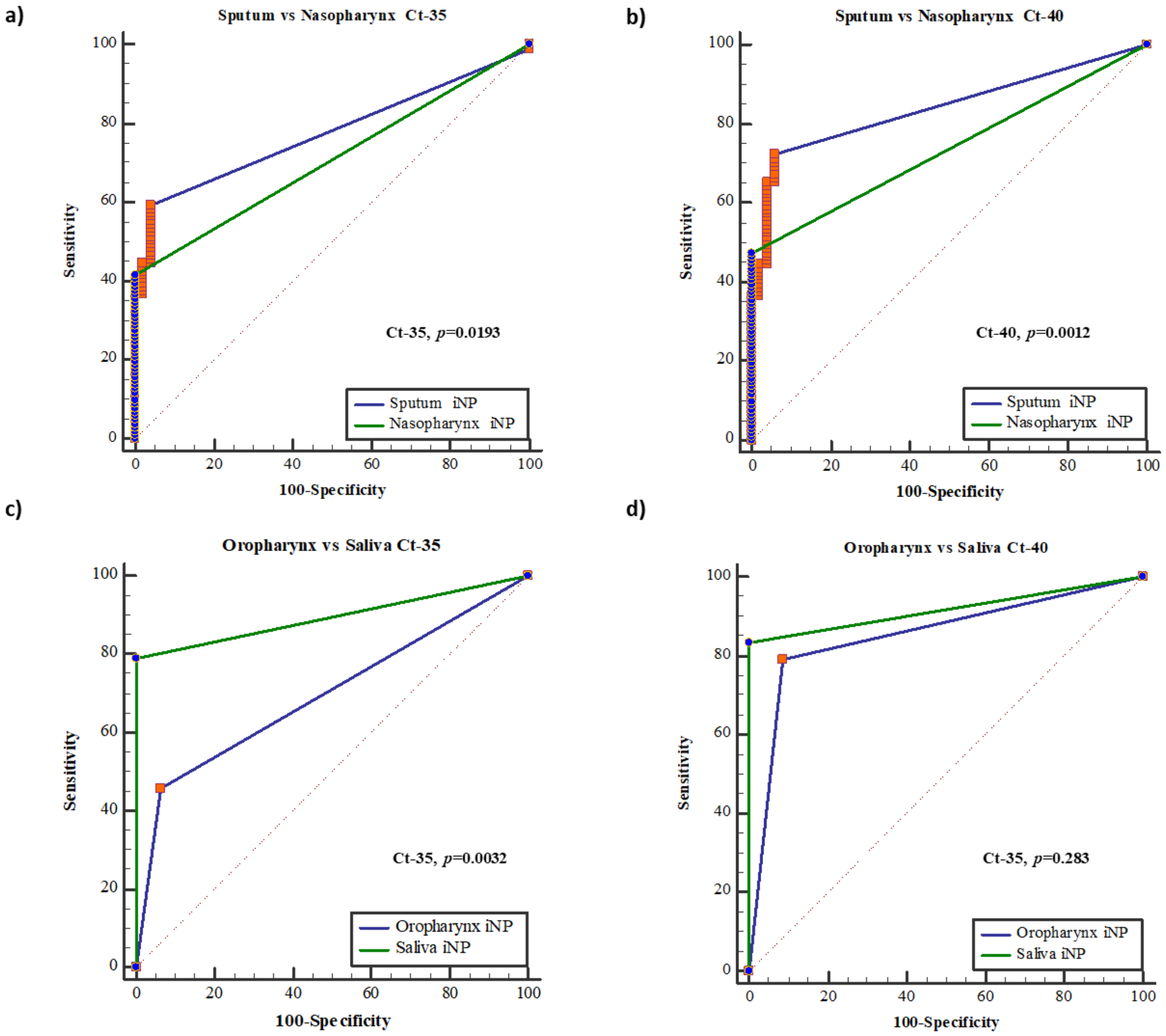

Supplementary Figure 1

The specificity and sensitivity of RT-qPCR with the in-house–designed *NP* gene primer set (iNP) in nasopharyngeal vs. sputum samples and oropharyngeal vs. saliva samples.

a) Determination of specificity and sensitivity in selected sputum vs. nasopharyngeal samples at a C_t cutoff of 35 (C_t-35).
b) Determination of specificity and sensitivity in selected sputum vs. nasopharyngeal samples at a C_t cutoff of 40 (C_t-40).
c) Determination of specificity and sensitivity in selected (first week) oropharyngeal vs. saliva samples at a C_t cutoff of 35 (C_t-35).
d) Determination of specificity and sensitivity in selected (first week) oropharyngeal vs. saliva samples at a C_t cutoff of 40 (C_t-40).

Table S1. All primers, probe, and PCR conditions used in this study.

Primer source	Target gene	Primer name	Primer sequence	Target	PCR condition
**WHO primer**	**RdRp gene**	RdRP_SARSr-F2	GTGARATGGTCATGTGTGGCGG	Pan Sarbeco	50℃ for 10 min, 1 cycle for Reverse transcription. 95℃ 30sec for 1 cycle for pre incubation, 95℃ 5sec for and 57℃ for amplification and data detection for 45 cycles
		RdRP_SARSr-R1	CARATGTTAAASACACTATTAGCATA		
		RdRP_SARSr-P1	[TET]CCAGGTGGWACRTCATCMGGTGATGC[BHQ1]		
**CDC primer**	**N gene 1**	2019-nCoV_N1-F	GACCCCAAAATCAGCGAAAT	SARS-CoV2	
		2019-nCoV_N1-R	TCTGGTTACTGCCAGTTGAATCTG		
		2019-nCoV_N1-P	[FAM]ACCCCGCATTACGTTTGGTGGACC[BHQ1]		
**CDC primer**	**N gene 2**	2019-nCoV_N2-F	TTACAAACATTGGCCGCAAA		
		2019-nCoV_N2-R	GCGCGACATTCCGAAGAA		
		2019-nCoV_N2-P	[FAM]ACAATTTGCCCCCAGCGCTTCAG[BHQ1]		
**CDC primer**	**N gene 3**	2019-nCoV_N3-F	GGGAGCCTTGAATACACCAAAA		
		2019-nCoV_N3-R	TGTAGCACGATTGCAGCATTG		
		2019-nCoV_N3-P	[FAM]AYCACATTGGCACCCGCAATCCTG[BHQ1]		
**This study**	**iNP gene**	nCov-NP_572F	GCAACAGTTCAAGAAATTC	SARS-CoV2	59℃ for amplification and the rest is same like above
		nCov-NP_687R	CTGGTTCAATCTGTCAAG		
		nCov-NP_661P	[FAM]AAGCAAGAGCAGCATCACCG[BHQ1]		
**Kogene Kit**	**E gene**	Primer/Probe mix 1 (E-gene)	Manufacturer's trade mark	SARS-CoV2	As per Manufacturer's instructions
	**RdRp gene**	Primer/Probe mix 2 (RdRp gene)	Manufacturer's trade mark	SARS-CoV2	As per Manufacturer's instructions

Table S2. Diagnostic characteristics of negative control samples.

Diagnostic characteristics	Negative samples			
	**Nasopharynx**	**Sputum**	**Oropharynx**	**Saliva**
No. of samples	55	21	24	28
NP-gene (iNP) RT-PCR	ND	ND	ND	ND
E-gene (Kogene kit) RT-PCR	ND	ND	ND	ND
RdRp-gene (Kogene kit) RT-PCR	ND	ND	ND	ND
WHO RdRp Primers RT-PCR	ND	ND	ND	ND
CDC N1 primers RT-PCR	ND	ND	ND	ND
CDC N2 primers RT-PCR	2 (+)	2 (+)	1 (+)	1 (+)
CDC N3 primers RT-PCR	ND	ND	ND	ND

Fifty-five nasopharyngeal, 21 sputum, 24 oropharyngeal, and 28 saliva samples were obtained from health checkup subjects with no clinical symptoms, no increase in a SARS-CoV-2 antibody titer, or no contact with confirmed COVID-19 patients. Cycle threshold >35 indicates a negative result; ND: not detectable; 2(+): two SARS-CoV-2–positive samples; 1(+): one virus-positive sample

Table S3. Clinical and subclinical characteristics of all COVID-19 patients

Patient	Gender/ Age	Underlying Comorbidity	Symptoms & signs	IFA		ELISA			Cell culture
				IgG titer Initial/ Follow-up	IgM titer Initial/ Follow-up	IgM antibody titer Initial/ Follow-up	IgG antibody titer Initial/ Follow-up	Total antibody titer Initial/ Follow-up	
**1**	M/46	HTN, dyslipidemia	coughing, chill	<1:32/1:256	<1:32/<1:32	<1:128/1:512	<1:128/1:4096	<1:128/1:4096	(+)
**2**	M/30	HTN	coughing, sore throat chill	<1:32/1:128	<1:32/<1:32	<1:128/<1:128	<1:128/1:512	<1:128/1:512	(+)
**3**	F/79	HTN,DM, dyslipidemia, Guillain-Barre syndrome	dyspnea	<1:32/1:1,024	<1:32/<1:32	<1:128/1:512	<1:128/1:2048	<1:128/1:2048	(+)
**4**	M/30	None	febrile sense	<1:32/1:128	<1:32/<1:32	<1:128/1:128	<1:128/1:1024	<1:128/1:1024	(+)
**5**	F/29	None	sore throat, myalgia chill	<1:32/1:64	<1:32/<1:32	<1:128/<1:128	<1:128/1:128	<1:128/<1:128	(+)
**6**	M/74	HTN,DM, dyslipidemia	sore throat, rhinorrhea	1:256/1:1,024	<1:32/<1:32	1:128/1:256	1:512/1:8192	1:512/1:4096	(-)
**7**	F/75	HTN, dyslipidemia	fever, sore throat	<1:32/>1:1,024	<1:32/1:32	<1:128/1:128	<1:128/1:1024	<1:128/1:1024	(-)
**8**	M/79	HTN,DM	fever, headache	<1:32/1:128	<1:32/<1:32	<1:128/1:256	<1:128/1:8192	<1:128/1:4096	(-)
**9**	F/61	None	fever, coughing	<1:32/1:256	<1:32/<1:32	<1:128/1:1024	1:256/1:16384	1:128/1:8192	(-)
**10**	M/36	None	sore throat, headache chill	<1:32/1:128	<1:32/<1:32	<1:128/<1:128	<1:128/1:128	<1:128/1:128	(-)
**11**	F/29	None	No symptoms	1:64/1:64	<1:32/<1:32	<1:128/<1:128	<1:128/1:128	<1:128/<1:128	(-)
**12**	M/22	None	No symptoms	1:64/1:64	<1:32/<1:32	<1:128/<1:128	<1:128/1:128	<1:128/1:128	(-)

HTN, hypertension; DM, diabetes mellitus. The IFA titer was measured by serial dilution of a patient's serum, and the IgG titer cutoff was ≥1:32. Antibody titer detection was performed (with serial dilution) by an indirect ELISA at OD_{450} against a recombinant SARS-CoV-2 nucleoprotein; the cutoff titer for IgM was 0.5; for IgG, it was 1.1; and for total Ig, it was 0.7

Table S4. RT-PCR results on SARS-CoV-2 in all clinical samples for primer set iNP and primer sets targeting the *E* and *RdRp* genes (Kogene Kit).

	**Specimens and values**	**Nasopharynx (N=101)**	**Oropharynx (N=90)**	**Sputum (N=106)**	**Pla/Ser/WB (N=77)**	**Saliva (N=69)**	**Stool (N=55)**	**Urine (N=92)**
**NP-gene (iNP)**	Positive test results, No%	42 (44.68%)	21 (23.59%)	61 (57.54%)	5 (6.32%)	32(46.37%)	6 (10.90%)	1 (1.08%)
	Cycle threshold, Mean (SD)	26.13 (13.5)	29.39 (4.4)	27.26 (5.1)	30.39 (3.01)	28.47 (4.1)	29.62 (3.5)	NA
	Ct range	10.04 - 34.94	14.94 - 33.96	14.94 -33.96	27.49 - 33.91	21.60 – 34.69	25.34 – 34.15	NA
	Sensitivity/Specificity	43.1%/100%	24.3%/100%	58.1%/100%		43.3%/100%		
	95% CI	0.628 - 0.779	14.77 - 32.95	0.709 - 0.841	2.56 - 14.08	0.644 - 0.808	3.64 - 20 .0	NA
	**Specimens and values**	**Nasopharynx (N=97)**		**Sputum (N=104)**				
**E-gene (Kogene kit)**	Positive test results, No%	38 (39.17%)		49 (47.11%)				
	Cycle threshold, mean (SD)	25.39 (5.2)		27.83 (5.0)				
	Ct range	12.88 – 34.33		13.76 - 34.93				
	Sensitivity/Specificity	36.5%/100%		46.1%/100%				
	95% CI	0.593 - 0.750		0.638 - 0.784				
	**Specimens and values**	**Nasopharynx (N=97)**		**Sputum (N=104)**				
**RdRp-gene (Kogene kit)**	Positive test results, No%	37 (38.18%)		47 (45.19%)				
	Cycle threshold, Mean (SD)	25.62(5.2)		27.96 (5.0)				
	Ct range	13.40 - 33.68		14.17 – 34.68				
	Sensitivity/Specificity	36.1%/100%		46.6%/100%				
	95% CI	0.593 - 0.750		0.652 - 0.796				

RT-PCR results on SARS-CoV-2 in all clinical samples collected from symptom onset to post-recovery, up to 5 weeks or more. Pla/Ser/WB: plasma/serum/whole blood; NA: not applicable; *N*: number; SD: standard deviation; CI: 95% confidence interval

Table S5. Sensitivity of RT-PCR with the in-house–designed *NP* gene primer set (iNP) in other samples depending on the time interval after symptom onset.

Days Since Symptoms	NP-gene (iNP)									
	Oropharynx (N=90)		Saliva (N=67)		WB/Plasma (N=77)		Urine (N=92)		Stool (N=55)	
	Ct < 35	Ct < 40	Ct < 35	Ct < 40	Ct < 35	Ct < 40	Ct < 35	Ct < 40	Ct < 35	Ct < 40
**Admission date** Sensitivity	50.0% (N =12)	83.3% (N =12)	70% (N =10)	80.0% (N =10)	12.5% (N =8)	37.5% (N =8)	0% (N =10)	10.0% (N =10)	0% (N =5)	20.0% (N =5)
**0 – 3 days** Sensitivity	35.7% (N =14)	64.3% (N =14)	69.2% (N =13)	69.2% (N =13)	18.2% (N =11)	27.3% (N =11)	0% (N =8)	12.5% (N =8)	0% (N =6)	16.7% (N =6)
**1 week(0 - 7 days)** Sensitivity	50.0% (N =26)	84.6% (N =26)	79.1% (N =24)	83.3% (N =24)	10% (N =20)	20.0% (N =20)	0% (N =22)	4.6% (N =22)	6.67% (N =15)	20.0% (N =15)
**2 weeks(8 – 14 days)** Sensitivity	20% (N =25)	40.0% (N =25)	37.5% (N =24)	62.5% (N =24)	12.5% (N =16)	31.3% (N =16)	3.83% (N =28)	15.4% (N =28)	14.3% (N =28)	31.3% (N =28)
**3 weeks(15 – 21 days)** Sensitivity	5.88% (N =17)	23.5% (N =17)	27.27% (N =11)	54.6% (N =11)	6.3% (N =16)	31.3 % (N =16)	0% (N =17)	23.5% (N =17)	14.3% (N =14)	31.3% (N =14)
**4 weeks(22 – 29 days)** Sensitivity	0% (N =8)	37.5% (N =8)	0% (N =4)	25.0% (N =4)	25.0% (N =4)	25.0% (N =4)	0% (N =8)	12.5% (N =8)	0% (N =4)	25.0% (N =4)
**5 weeks+(30 + days)** Sensitivity	9.1% (N =11)	27.3% (N =11)	0% (N =3)	33.3% (N =3)	0% (N =22)	13.6% (N =22)	0% (N =20)	10.0% (N =20)	0% (N =9)	13.6% (N =9)

Samples were segregated as follows: admission date, 0–3 days after admission, 0–7 days after admission as 1 week, 8–14 days as 2 weeks, 15–21 days as 3 weeks, 22–29 as 4 weeks, and 30+ as 5 weeks+. *N*: number of samples; WB: whole blood
